# Evaluating the Source and Content of Internet Information Regarding DeQuervain’s Tenosynovitis

**DOI:** 10.7759/cureus.70882

**Published:** 2024-10-05

**Authors:** Kyle Plusch, Daniel Z Givner, Jack Carfagno, Moody Kwok, Pedro Beredjiklian

**Affiliations:** 1 Hand Surgery, Rothman Orthopaedic Institute, Philadelphia, USA; 2 Orthopaedic Surgery, Sidney Kimmel Medical College, Philadelphia, USA; 3 Orthopaedic Surgery, Philadelphia College of Osteopathic Medicine, Philadelphia, USA

**Keywords:** content, dequervain's tenosynovitis, information, internet, quality

## Abstract

Introduction

Patients are increasingly relying on the internet for information about their medical diagnoses, but there is little regulation on the content of these websites. The purpose of this study was to investigate the quality of the informational content available on the internet regarding DeQuervain’s tenosynovitis.

Methods

The search phrasing “‘DeQuervain’ OR ‘DeQuervain’s’ OR ‘Radial styloid tenosynovitis’ OR ‘DeQuervain’s synovitis’ OR ‘Wrist tendonitis’” was entered into the five most commonly used search engines, and the first 50 universal resource locators (URLs) were recorded, including sponsored sites. Duplicate sites and URLs linking to other search engines were removed, yielding a roster of 109 websites that form the basis for this study. Each site was graded by two authors and scored based on a previously published information evaluation protocol regarding informational content. A third individual resolved any conflict before analysis. The sum of these scores resulted in an “Informational Score” on a scale from 0 to 100.

Results

Sixty-six (60.6%) of the websites evaluated were authored by an academic institution or a physician, and these all provided conventional information. Thirty-two (29.4%) of the sites were commercial sites or sold commercial products. The remaining 11 websites (10.1%) had nonphysician, unidentified, or lay authorship. The mean informational score for all sites was 46.7 out of a maximum of 100 points. The mean score of the academic or physician-authored websites was 53.3 out of 100 points, compared to 36.7 out of 100 for the remainder of the sites, which was significantly different. The mean score for the 14 sponsored websites was 11.6 out of 100.

Discussion/conclusion

We concluded that internet information on DeQuervain’s tenosynovitis is of poor quality and incomplete. Patients looking for information about DeQuervain’s tenosynovitis on the internet should be advised to focus on academic- and physician-authored sites in order to avoid commercial bias and misleading information.

## Introduction

DeQuervain’s tenosynovitis was initially discovered in 1895 by the Swiss surgeon Dr. Friz de Quervain [1,2]. It results in the thickening of the tendon sheaths around the abductor pollicis longus (APL) and extensor pollicis brevis (EPB) tendons at the styloid process of the radius [2-4]. As a result of this thickening, pain occurs at the radial wrist due to the resisted gliding of the APL and EPB tendons in the fibro-osseous canal. This pain is exacerbated by thumb, radial, or ulnar movements [4]. The condition is caused by overuse or repetitive movements of the tendons in the first dorsal compartment [4]. Treatment ranges from conservative treatment of resting, icing, and bracing the wrist to surgical interventions [3].

The origin of the internet dates back to the 1960s, yet it was not made public until August 6, 1991 [5,6]. At that time, there was only a single website created, and roughly 0.4% of the world had access to the internet. Since then, over a trillion websites have been created and, roughly 65.6% of the world has access to it today [7]. With the increasing frequency of websites being created, more information as well as misinformation can be spread across the globe. According to Reuter’s report, roughly 66% of patients look online for health and medical information and they have access to roughly 10,000 medically related websites after a few clicks [8]. Specifically, in orthopedics, a report by Burrus et al. demonstrated that 64.7% of orthopedic patients utilized the internet to access health information regarding their condition and treatment [9]. With the oversaturation of healthcare websites, the quality of information varies tremendously from website to website. The goal of this study was to evaluate the informational quality of the content regarding DeQuervain’s tenosynovitis on the internet. We hypothesized that the information on the internet would provide adequate information based on our current understanding of the pathophysiology and treatment of this condition.

This article was previously presented as a meeting abstract at the 2023 American Association for Hand Surgery Annual Meeting on January 17-21, 2023.

## Materials and methods

The study mimicked previous study designs evaluating the quality of medical information on the internet [5]. The phrasing “‘DeQuervain’ OR ‘DeQuervain’s’ OR ‘Radial styloid tenosynovitis’ OR ‘DeQuervain’s synovitis’ OR ‘Wrist tendonitis’” was used in order to mimic the different ways that patients may search for information on the disease. This search was entered into the five most commonly used English search engines (Google [www.google.com], Bing [www.bing.com], Yahoo [www.yahoo.com], AOL [www.aol.com], and Ask [www.ask.com]) [10]. On each search engine, the first 50 universal resource locators (URLs) were recorded, including sponsored sites, which were noted. A total of 250 sites were filtered to remove duplicates and links to other search engines, resulting in a final list of 109 websites for informational scoring. A previously published information evaluation protocol developed by Soot et al. was used to grade each website [11].

Each site was graded according to two of the authors and scored based on authorship, content, disease summary, treatment options, pathogenesis, complications of treatment, and results of treatment. Any conflict on authorship or content was resolved by a third author [5]. The “Informational Value” final score is the sum of the disease summary, treatment options, pathogenesis, complications, and results, ranging from 0 to 100 [5].

Authorship

The primary author of the website information was evaluated to fall into one of the following seven categories: (1) "academic" indicated a stated affiliation with a university or research organization; (2) "physician" indicated that the author(s) were physicians in individual or group-practice who were not affiliated with a university or research organization or whose affiliation was not stated; (3) "nonphysician" care provider indicated alternative care providers including chiropractors, physical/occupational therapists, or acupuncturists; (4) "commercial site" indicated that the author represented a commercial website without an interest in a specific commercial product (typically, the stated purpose of these websites was to provide medical information to the lay public); (5) "commercial product" indicated an author or authors who were marketing a commercial product for treatment or evaluation of DeQuervain’s disease; (6) "lay" indicated identifiable organizations or individuals who did not belong to any of the previous categories and who authored a noncommercial website for providing information about DeQuervain’s disease; or (7) "unidentified" indicated that the author was not identifiable on the website [5].

Content

The informational content regarding evaluation, treatment, pathogenesis, and prevention of DeQuervain’s tenosynovitis on each site was described according to one of four categories: (1) "conventional" indicated that the site provided information consistent with evidence-based knowledge based on orthopedic literature; (2) "unconventional" indicated that the site provided additional alternative information that was not evidence-based in addition to conventional knowledge without evidence of commercial bias; (3) "misleading" indicated that the site offered unconventional information with clear evidence of bias for commercial gains; or (4) "noninformational" indicating that the site did not contain patient-related information [5].

Informational value

Disease Summary (Maximum, 30 Points)

Three points each were awarded when any of the following 10 factors were mentioned or clearly described: pain, stiffness, weakness, swelling, anatomy of the wrist, snapping sensation, decreased strength on physical examination, decreased motion on physical examination, discussion of progression of disease, and diagnosis with imaging [5].

Treatment Options (Maximum, 20 Points)

Five points each were awarded when any of the following treatment options were given: splinting, oral anti-inflammatory medications, activity modification, corticosteroid injections, and surgery.

*Pathogenesis (Maximum, 20 Points)*

Five points were awarded for each of the following etiologies mentioned: trauma, pregnancy, child caretaking, overuse, and idiopathic.

Complications of Treatment (Maximum, 15 Points)

Seven and one half (7.5) points were awarded for each of the following categories mentioned: complications of nonoperative treatment (including disease progression, side effects of corticosteroid injections, or oral anti-inflammatory medication) and complications of operative treatment (such as stiffness, infection, nerve injury, or progression of disease). Any single mention of a complication in each category was sufficient to earn a full 7.5 points.

*Results of Treatment (Maximum, 15 Points)*

Seven and one half (7.5) points were awarded when the results of nonoperative treatment were given, and 7.5 points were awarded when the results of operative treatment were given. Similarly, a single mention of results in each category was sufficient to earn a full 7.5 points.

Statistical analysis was performed using the Student’s t-test for continuous variables. Interobserver reliability was calculated by comparing agreement rates between authorship/content type and overall informational scores. Each site’s total informational score used for analysis is the average of the two authors’ grades for each site.

## Results

With regard to the authorship of the websites, 66 (60.6%) were authored by an academic institution or physician. Seven (6.4%) were authored by a nonphysician care provider, 17 (15.6%) were part of a commercial site, 15 (13.8%) were solely advertising a commercial product, and four (3.7%) were unidentified. With regard to website content, 86 (78.9%) of all sites provided conventional information. six (5.5%) provided unconventional information, 10 (9.2%) were misleading, and seven (6.4%) were noninformational. Notably, all 66 academically affiliated or physician-authored sites provided conventional information; four out of seven nonphysician-authored sites and 14 out of 17 commercial sites offered conventional information. All 15 sites promoting commercial products were either misleading or noninformational. A full breakdown of content and authorship is shown in Table 1. 

**Table 1:** Authorship and Content of Websites

	Academic	Physician	Nonphysician	Commercial site	Commercial product	Lay	Unidentified	Total
Overall	30	36	7	17	15	0	4	109
Conventional	30	36	4	14	0	0	2	86
Unconventional	0	0	2	3	0	0	1	6
Misleading	0	0	1	0	9	0	0	10
Noninformational	0	0	0	0	6	0	1	7

The mean total informational score for all 109 unique websites was 46.7 out of a possible 100. The mean scores for each specific content section are as follows: disease summary, 11.1 out of 30; treatment options, 15 out of 20; pathogenesis, nine out of 20; complications of treatment, 4.2 out of 15; and results of treatment, 7.4 out of 15. The mean informational score of academic and physician-authored sites was 53.3 compared to 36.7 for the rest of the authorship categories; this difference is statistically significant (p<0.001). Within each subcategory, academic/physician-authored sites had the highest average score in all categories. The 109 unique sites included 14 sponsored websites; excluding the sponsored sites, the mean total informational score was 51.9, and the mean score for the sponsored sites was 11.6. A full breakdown of the informational score for each authorship category is shown in Table 2.

**Table 2:** Informational Scores of Websites

	Disease summary	Treatment options	Pathogenesis	Complications of treatment	Results of treatment	Total
All sites	11.1	15.0	9.0	4.2	7.4	46.7
All sites excluding advertisements	12.2	16.6	9.9	4.8	8.4	51.9
Academic/physician-authored only	12.4	16.7	9.9	5	9.3	53.3
Excluding academic/physician-authored	9.2	12.3	7.7	3.1	4.4	36.7
Advertisement	4.2	3.8	2.9	0.5	0.3	11.6

Regarding interobserver reliability, the mean overall average score differed by 5.7% between the two observers, indicating a high degree of reliability. Eighteen sites (17%) had authorship conflicts and 11 (10%) had content conflicts, which were all resolved by a third author before analysis.

## Discussion

Our review of the top 50 search results on the five most commonly used search engines resulted in 109 unique websites for grading. With an average total informational score of 46.7 out of 100, we conclude that the quality of internet information on DeQuervain’s tenosynovitis is of poor quality. Academic and physician-authored sites scored the highest in each category of our grading system compared with other sites. Despite these better results, overall scoring fell below 50% in many categories. These higher-quality sites only averaged five out of 15 points for discussing complications and 12.4 out of 30 for “disease summary.” The lack of complete information on treatment and complications is consistent with a study from Anderson et al. in 2014 regarding a surgical implant for neurogenic claudication; they found that only 23% of websites directly mentioned complications, which is even more vital when discussing a specific surgical treatment [12]. Websites related to femoroacetabular impingement only reported complications of treatment 27% of the time and only mentioned treatment options 54% of the time [13].

A previous study scoring websites related to Dequervain’s tenosynovitis from Heap et al. in 2015 found an average quality score of 20.3 out of 30, based on their 30-point grading system [14]. Their percentage of maximum score is slightly higher than we found (60% vs. 47%); however, many of their scoring points for grading were more general criteria contrary to our specific criteria, which likely led to more lenient scoring. A recent study from Katt et al. in 2021 on the quality of information on Kienbock’s disease used the same grading scale as in this study and yielded a very similar average total score of 45.5 out of 100 [15]. While the sample size was smaller (49 sites), their breakdown of authorship and content was similar to ours, including the significant difference in quality between academic/physician-authored sites compared to all others. Our average quality score of 47% was similar to numerous other orthopedic-related internet information studies using a variety of scoring systems, including shoulder arthritis (46%), lateral epicondylitis (40%), hallux valgus (42%), acetabular fractures (41%), and pelvic fractures (42%) [16-19]. One commonly used scale is the DISCERN score, which consists of 16 questions each graded with a five-point Likert scale for a maximum score of 80 [20]. Other studies frequently assess the “readability” of websites; the recommended reading level for patient material is eighth grade or lower, yet the majority of websites fail to meet these criteria [19,21-23], resulting in websites that are both incomplete with regard to information and difficult to comprehend by an average patient. The Health On the Net (HON) code of conduct system represents another way to identify if healthcare information on the internet is reliable; multiple studies have shown that websites with a HON seal and higher HON scores also had higher content scores [24,25]. Unfortunately, this system is infrequently utilized; a prior study on trapeziometacarpal arthritis websites found only three out of 60 websites contained a HON code [26]. Despite a wide variety in the various grading systems, the overall average scores of internet quality studies consistently fall below par. 

All five search engines displayed “sponsored” websites throughout their pages; these typically have a small “Ad” marking next to the URL, appear at the top of the page of results (although occasionally at the bottom), and are paid to be present in the search results by the website that they link to. The majority of other internet information quality studies exclude these sites in their analysis. We chose to include them based on their prevalence among all of the search engines as well as the fact that an internet user may assume that they are of high quality based on their placement at the top of the search result page. The “ad” designation is quite small and could easily be missed by a patient. Of the initial 250 total URLs, 84 were sponsored advertisements. The majority of these were repeated across multiple search engines, and many linked to a new search engine. After these were excluded, 14 were included for final analysis. Of the 14 sponsored sites, 11 (79%) were advertising solely for a commercial product, and 12 (86%) were either noninformational or provided misleading information, leading to a low informational score average of 11.6. This data is very similar to the findings of Lutsky et al, in 2013; they looked at the top five sponsored sites over five search engines for carpal Tunnel Syndrome using the same grading scale as in this study [27]. They found an average score of 14.5, 19 out of 25 (76%) advertising a commercial product, and 19 out of 25 (76%) were noninformational or provided misleading information. This indicates that the quality of sponsored websites on search engines has not improved significantly over the years.

The grading scale used in this study comes with some limitations. First, there is a lack of objective accuracy measurement. A website that mentions all of the possible grading points on our scale, while also mentioning other extraneous incorrect statements, would receive a higher score than a website that mentioned fewer points but with 100% accuracy. However, the use of specific objective criteria in our grading based on factual information about DeQuervain’s disease ensured that there were accurate statements made in order to score points. Additionally, we included articles that required a subscription or payment to access the full text, grading only what was visible for free (for example, a Medscape article or a peer-reviewed publication, where the full text required a subscription). We wanted to be as inclusive as possible with the websites included for analysis, assuming that an average patient would open the article but not necessarily have full access. Therefore, these articles resulted in a much lower score than if they were graded based on their full-access text. In a similar situation, we did not follow any links on any websites, only grading information directly visible on the page. Some websites may have had additional information buried under multiple pages within one overall site, and the results may have varied with how thoroughly a patient would search through a website.

## Conclusions

Based on our findings, patients looking for information about DeQuervain’s tenosynovitis on the internet should be advised to focus on academic and physician-authored sites. While individual sites are usually not a complete source of information, viewing multiple academic or physician-authored sites will provide the highest information quality while avoiding commercial bias and misleading information. As internet usage undoubtedly continues to expand, encouraging online literacy skills with patients will prove vital in preventing the dissemination of misleading or incomplete information. The ability to understand when a site is actually a paid advertisement and identify the type of authorship on a webpage would allow a patient to quickly filter out low-quality search results. Providing patients with informational material created by the physician or office staff is one way to ensure that patients receive only factual information about their condition; however, with the current prevalence of internet usage, it cannot be assumed that patients will not search for additional information on the internet. Therefore, the owners of websites dedicated to medical information should continue to work to ensure that their information is stated completely and accurately.
